# Quorum and Light Signals Modulate Acetoin/Butanediol Catabolism in *Acinetobacter* spp.

**DOI:** 10.3389/fmicb.2019.01376

**Published:** 2019-06-20

**Authors:** Marisel Romina Tuttobene, Laura Fernández-García, Lucía Blasco, Pamela Cribb, Anton Ambroa, Gabriela Leticia Müller, Felipe Fernández-Cuenca, Inés Bleriot, Ramiro Esteban Rodríguez, Beatriz G. V. Barbosa, Rafael Lopez-Rojas, Rocío Trastoy, María López, Germán Bou, María Tomás, María A. Mussi

**Affiliations:** ^1^Centro de Estudios Fotosintéticos y Bioquímicos de Rosario (CEFOBI-CONICET), Facultad de Ciencias Bioquímicas y Farmacéuticas, Universidad Nacional de Rosario, Rosario, Argentina; ^2^Microbiology Department-Biomedical Research Institute A Coruña (INIBIC), Hospital A Coruña (CHUAC), University of A Coruña (UDC), A Coruña, Spain; ^3^Instituto de Biología Molecular y Celular de Rosario (IBR-CONICET), Rosario, Argentina; ^4^Clinical Unit for Infectious Diseases, Microbiology and Preventive Medicine, Hospital Universitario Virgen Macarena, Seville, Spain; ^5^Department of Microbiology and Medicine, University of Seville, Seville, Spain; ^6^Biomedicine Institute of Seville (IBIS), Seville, Spain; ^7^Microbial Resistance Laboratory, Biological Sciences Institute, University of Pernambuco (UPE), Recife, Brazil

**Keywords:** acetoin, BLSA, AcoN, light, *Acinetobacter*

## Abstract

*Acinetobacter* spp. are found in all environments on Earth due to their extraordinary capacity to survive in the presence of physical and chemical stressors. In this study, we analyzed global gene expression in airborne *Acinetobacter* sp. strain 5-2Ac02 isolated from hospital environment in response to quorum network modulators and found that they induced the expression of genes of the acetoin/butanediol catabolism, volatile compounds shown to mediate interkingdom interactions. Interestingly, the *acoN* gene, annotated as a putative transcriptional regulator, was truncated in the downstream regulatory region of the induced acetoin/butanediol cluster in *Acinetobacter* sp. strain 5-2Ac02, and its functioning as a negative regulator of this cluster integrating quorum signals was confirmed in *Acinetobacter baumannii* ATCC 17978. Moreover, we show that the acetoin catabolism is also induced by light and provide insights into the light transduction mechanism by showing that the photoreceptor BlsA interacts with and antagonizes the functioning of AcoN in *A. baumannii*, integrating also a temperature signal. The data support a model in which BlsA interacts with and likely sequesters AcoN at this condition, relieving acetoin catabolic genes from repression, and leading to better growth under blue light. This photoregulation depends on temperature, occurring at 23°C but not at 30°C. BlsA is thus a dual regulator, modulating different transcriptional regulators in the dark but also under blue light, representing thus a novel concept. The overall data show that quorum modulators as well as light regulate the acetoin catabolic cluster, providing a better understanding of environmental as well as clinical bacteria.

## Introduction

*Acinetobacter baumannii* has recently been recognized by the World Health Organization (WHO) as one of the most threatening pathogens deserving urgent action (Tacconelli et al., 2018). With the aid of new taxonomic tools and technological advancements, other members of the *Acinetobacter* genus have also been identified as causative agents of hospital acquired infections and are gaining clinical relevance (Turton et al., 2010; Karah et al., 2011). Key factors determining their success as pathogens include their extraordinary ability to develop resistance to antimicrobials as well as to persist in the hospital environment despite adverse conditions such as desiccation, lack of nutrients, etc. (McConnell et al., 2013; Spellberg and Bonomo, 2014; Yakupogullari et al., 2016). It is known that some members of the genus can be transmitted by air. In fact, some genotypes of *A. baumannii* have been shown to survive for up to 4 weeks in the air in intensive care units (ICUs) (Yakupogullari et al., 2016). It is becoming increasingly clear, despite not very much studied, the importance of this kind of transmission since it leads to recontamination of already decontaminated surfaces, transmission between patients, airborne contamination of healthcare providers as well as of medical instruments (Spellberg and Bonomo, 2013). We have recently reported the genome sequence of *Acinetobacter* sp. strain 5-2Ac02 (closely related to *Acinetobacter towneri*), which has been recovered from the air in an ICU of a hospital in Rio de Janeiro, Brazil (Barbosa et al., 2016). This strain was shown to harbor a much reduced genome and higher content of insertion sequences than other *Acinetobacter* sp. Moreover, four different toxin–antitoxin (TA) systems as well as heavy metal resistance operons were found encoded in its genome (Barbosa et al., 2016). Interestingly, some bacteria have been shown to produce and release a large diversity of small molecules, including organic and inorganic volatile compounds such as acetoin and 2,3-butanediol (BD), referred as bacterial volatile compounds (BVCs), which can mediate airborne bacterial interactions (Audrain et al., 2015). BVCs can mediate cross-kingdom interactions with fungi, plants, and animals, and can even modulate antibiotic resistance, biofilm formation, and virulence (Audrain et al., 2015).

Several molecular mechanisms have been associated with the development of bacterial tolerance or persistence under stress conditions (environmental or drug-related) (Trastoy et al., 2018). Among these are included the general stress response (RpoS-mediated), tolerance to reactive oxygen species (ROS), energy metabolism, drug efflux pumps, the SOS response, and TA systems, with the quorum network (quorum sensing/quorum quenching) regulating many of them (Trastoy et al., 2018). The finding that many bacterial pathogens are able to sense and respond to light modulating diverse aspects related to bacterial virulence and persistence in the environment is particularly pertinent in this context. Indeed, light has been shown to modulate biofilm formation, motility, and virulence against *C. albicans*, a microorganism sharing habitat with *A. baumannii*, at environmental temperatures in this pathogen. Moreover, light modulates metabolic pathways including trehalose biosynthesis and the phenylacetic acid degradation pathway, antioxidant enzyme levels such as catalase, and susceptibility or tolerance to some antibiotics (Ramirez et al., 2015; Muller et al., 2017). In addition, light induced the expression of whole gene clusters and pathways, including those involved in modification of lipids, the complete type VI secretion system (T6SS), acetoin catabolism, and efflux pumps (Muller et al., 2017). Many of these processes are controlled by BlsA, the only canonical photoreceptor codified in the genome of *A. baumannii*, which is a short blue light using flavin (BLUF) protein. BlsA has been shown to function at moderate temperatures such as 23°C but not at 37°C by a mechanism that includes control of transcription as well as photoactivity by temperature (Mussi et al., 2010; Abatedaga et al., 2017; Tuttobene et al., 2018). Knowledge of these mechanisms will potentially enable the implementation of several clinical or industrial applications.

In this study, we characterized the airborne *Acinetobacter* sp. strain 5-2Ac02, analyzing gene expression adjustments in response to environmental stressors such as mitomycin C and acyl-homoserine-lactones, which modulate the quorum network. The results showed that genes involved in the SOS response, TA systems, and heavy metal resistance were induced in response to mitomycin, while genes involved in acetoin and aromatic amino acid catabolism were modulated as a response to quorum sensing signals. The fact that acetoin catabolic genes were also found to be induced by light in *A. baumannii* (Muller et al., 2017) prompted us to deepen the study on this metabolism. In bacteria, the butanediol fermentation is characterized by the production of BD and acetoin from pyruvate. The production of butanediol is favored under slightly acidic conditions and is a way for the bacteria to limit the decrease in external pH caused by the synthesis of organic acids from pyruvate. The catabolic α-acetolactate-forming enzyme (ALS) condenses two molecules of pyruvate to form one α-acetolactate, which is unstable and can be converted to acetoin by α-acetolactate decarboxylase (ALDC) or diacetyl as a minor by-product by non-enzymatic oxidative decarboxylation. Diacetyl can be irreversibly transformed into its reductive state acetoin, and acetoin can be reversibly transformed into its reductive state BD, both catalyzed by 2,3-butanediol dehydrogenase (BDH). The acetoin breakdown in many bacteria is catalyzed by the acetoin dehydrogenase enzyme system (AoDH ES), which consists of acetoin:2,6-dichlorophenolindophenol oxidoreductase, encoded by *acoA* and *acoB*; dihydrolipoamide acetyltransferase, encoded by *acoC*; and dihydrolipoamide dehydrogenase, encoded by *acoL* (Xiao and Xu, 2007). Our results show that the *acoN* gene codes for a negative regulator of the acetoin/butanediol catabolic cluster and is involved in photoregulation of acetoin catabolism in *A. baumannii* through the BlsA photoreceptor. Most importantly, we provide strong evidence on the mechanism of light signal transduction, which is far from being understood for BlsA or other short BLUF photoreceptors, taking into account in addition that BlsA is a global regulator in *A. baumannii*. In this sense, we have recently shown that this photoreceptor binds to and antagonizes the functioning of the Fur repressor only in the dark at 23°C, presumably by reducing its ability to bind to acinetobactin promoters, thus relieving repression at the transcriptional level as well as growth under iron limitation at this condition (Tuttobene et al., 2018). Here, we further show that BlsA directly interacts with the acetoin catabolism negative regulator AcoN at 23°C but, in this case, in the presence of blue light rather than in the dark. In fact, growth on acetoin was much better supported under blue light than in the dark through BlsA and AcoN. Moreover, acetoin catabolic genes were induced at this condition in a BlsA- and AcoN-dependent manner. Opposite behavior was observed for *ΔblsA* and *ΔacoN* mutants, being BlsA necessary for the observed induction while AcoN for repression, thus indicating that BlsA antagonizes AcoN. Finally, yeast two-hybrid (Y2H) assays indicate that BlsA interacts with AcoN only under blue light but not in the dark. The results strongly suggest that BlsA interacts with and likely sequesters the acetoin repressor under blue light but not in the dark. Thus, in the presence of light, acetoin catabolic genes are relieved from repression resulting in much better bacterial growth in this condition. Here again, the phenomena depends on temperature, occurring at low–moderate temperatures such as 23°C but not at 30°C, consistent with previous findings of our group for BlsA functioning (Mussi et al., 2010; Abatedaga et al., 2017; Tuttobene et al., 2018).

## Materials and Methods

### Bacterial Strains, Plasmids, and Media

Bacterial strains and plasmids used in this work are listed in Table 1. Luria-Bertani (LB) broth (Difco) and agar (Difco) were used to grow and maintain bacterial strains. Broth cultures were incubated at the indicated temperatures either statically or with shaking at 200 rpm.

**TABLE 1:** Minimal inhibitory concentrations (MICs) of several antibiotics and heavy metals for *Acinetobacter* sp. strain 5-2Ac02.

**Antimicrobial**	**MIC (μg/mL)**	**Category^a^**
Sulbactam	0.5	Susceptible
Piperacillin	0.06	Susceptible
Ceftazidime	4	Susceptible
Imipenem	0.06	Susceptible
Meropenem	0.03	Susceptible
Doripenem	0.015	Susceptible
Ciprofloxacin	1	Susceptible
Amikacin	1	Susceptible
Gentamicin	0.25	Susceptible
Tobramycin	0.5	Susceptible
Netilmicin	0.25	Susceptible
Tetracycline	0.5	Susceptible
Minocycline	<0.002	Susceptible
Doxycycline	0.03	Susceptible
Tigecycline	0.25	Susceptible
Colistin	0.125	Susceptible
Clavulanic acid	4	Susceptible
Azithromycin	16	Susceptible
**Heavy metal**	**MIC (μg/mL)**	**Category^b^**
Arsenic	>2048	**Resistant**
Cadmium	64	Susceptible
Cobalt	16	Susceptible
Cooper	266	**Resistant**
Chromium	128	Susceptible
Tellurite	2	Susceptible
Zinc	256	Susceptible

*^a^MICs were determined by the microdilution method, in accordance with general procedures recommended by CLSI. For specific details, please refer to the section “Materials and Methods.” ^b^Determined according to Taylor et al. (2002) and Akinbowale et al. (2007). In bold are shown the resistance category.*

### Plasmid Construction

#### Y2H Plasmid Construction

PCR amplification of *blsA* and *acoN* coding sequences was performed from *A. baumannii* ATCC 17978 genomic DNA using primers blsAdh and acoNdh (Supplementary Table S2). The amplification products were subsequently cloned into the BamHI and XhoI sites of Gateway entry vector pENTR3C (Invitrogen) (Supplementary Table S1). The cloned fragments were then transferred to pGBKT7-Gw and pGADT7-Gw Y2H vectors (Clontech) by using LR Clonase (Cribb and Serra, 2009; Tuttobene et al., 2018). In the yeast host, these plasmids express the cloned coding sequences as fusion proteins to the GAL4 DNA-binding domain (DB) or activation domain (AD), respectively, under the control of the constitutive ADH1 promoter. Automated DNA sequencing confirmed correct construction of each plasmid.

#### pWHAcoN Plasmid Construction

*acoN* coding sequence and its promoter were amplified by PCR using *A. baumannii* ATCC 17978 genomic DNA as template and primers PAcoNF and PAcoNR (Supplementary Table S2), which contained BamHI restriction site tails. The amplification product was cloned into pWH1266 through the BamHI sites. Automated DNA sequencing confirmed the proper construction of pWHAcoN plasmid.

#### Susceptibility to Antimicrobials and Heavy Metals (MICs)

The antibiotic and heavy metal susceptibility profile by microdilution was determined according to CLSI recommendations (Table 1). Heavy metal susceptibility was determined by broth microdilution following CLSI instructions for cobalt, chromium, copper, arsenic, and zinc (Akinbowale et al., 2007). The susceptibility to tellurite was determined by serial plate dilution, with concentrations ranging from 1 to 1024 μg/mL *Escherichia coli* K12 w*ere* used as reference strain (Akinbowale et al., 2007). The breakpoints adopted for resistance phenotype were as follows: ≥100 μ*g*/mL for cadmium; ≥200 μ*g*/mL for copper, arsenic, and zinc; ≥400 μ*g*/mL for cobalt; ≥800 μ*g*/mL for chromium*;* and >128 μ*g*/mL for tellurite.

Growth curves in the presence of heavy metals were performed as follow: one colony of *Acinetobacter* sp. strain 5-2Ac02 was grown overnight, diluted 1:100 in 20 mL of low nutrient LB broth, and incubated at 37°C with shaking (180 rpm) (Lopez et al., 2018). The cultures were grown for 4 h to the exponential phase; and then, the heavy metals were added. For each isolate, the proportion of survivors was determined: (i) in the control without heavy metals, (ii) in the presence of arsenic (0.50× MIC), (iii) in the presence of cupper (0.5× MIC). Bacterial concentrations (log_10_ CFU/mL) were determined at 0, 2, 4, 24, and 48 h by serial dilution and plating on LB agar. All experiments were performed in duplicate.

#### Gene Expression by Microarrays Under Stress Conditions: Mitomycin and AHLs

*Acinetobacter* sp. strain 5-2Ac02 cells were grown in LB medium to an exponential phase about OD_600_ = 0.5 before addition of 10 μg/mL of mitomycin C (SOS response) or a mixture of 1 μM each acyl-homoserine lactones composed by *N*-(butyl, heptanoyl, hexanoyl, β-ketocaproyl, octanoyl, and tetradecanoyl)-DL-homoserine lactones or 10 μM 3-oxo-dodecanoyl-HSL (3-oxo-C12-HSL) (Quorum Network). After incubation of the mixtures for 2 h, 1 mL of each culture was used for RNA extraction. RNA was purified using the High Pure RNA Isolation Kit (Roche, Germany). The microarrays were specifically designed for this strain using eArray (Agilent). The microarray assays were performed with 12,664 probes to study 2,795 genes. Labeling was carried out by two-color microarray-based prokaryote analysis and Fair Play III labeling, version 1.3 (Agilent). Three independent RNAs per condition (biological replicates) were used in each experiment. Statistical analysis was carried out using Bioconductor, implemented in the RankProd software package for the R computing environment. A gene was considered induced when the ratio of the treated to the untreated preparation was 1.5 and the *p*-value was <0.05 (Lopez et al., 2017b).

#### Bacterial Killing Curves

The MICs of ampicillin, ciprofloxacin, and mitomycin C were determined for *Acinetobacter* sp. strain 5-2Ac02 (0.5, 1, and 0.5 μg/mL) versus *A. baumannii* strain ATCC 17978 (8, ≤0.12, and 2 μg/mL). Briefly, an initial inoculum of 5 × 10^5^ CFU/mL was incubated at 37°C with shaking (250 rpm) in 20 mL of low nutrient LB broth (LN-LB; 2 g/L tryptone, 1 g/L yeast extract, and 5 g/L NaCl) (Lopez et al., 2017a, 2017b). The cultures were grown for 4 h to the exponential phase; and then, the antibiotics were added. For each isolate, the proportion of survivors was determined: (i) in the control without antibiotic, (ii) in the presence of mitomycin C (0.25× MIC), (iii) in the presence of ampicillin (10× MIC), (iv) in the presence of ciprofloxacin (10× MIC), (v) in the presence of mitomycin C and ampicillin (0.25× MIC and 10× MIC), and (vi) in the presence of mitomycin C and ciprofloxacin (0.25× MIC and 10× MIC). Bacterial concentrations (log_10_ CFU/mL) were determined at 0, 1, 2, 3, 4, 20, 24, 28, and 48 h by serial dilution and plating on Mueller-Hinton agar. All experiments were performed in triplicate. This protocol was performed following previously described indications (Hofsteenge et al., 2013). Finally, the persister sub-population was determined from the percentage of survivors.

#### Gene Deletion in *A. baumannii* ATCC 17978

The negative regulator of the acetoin operon was deleted following the double recombination method, using the pMO-TelR plasmid and *E. coli* DH5α strain to multiply the plasmid with the construct (Hamad et al., 2009; Aranda et al., 2010). All primer sequences used were designed in this study and are listed in Supplementary Table S2.

#### Isolation of RNA and Analyses of Genes Expression by qRT-PCR

*Acinetobacter baumannii* cells were grown stagnantly in LN-LB at 37°C with the addition of 10 μM of 3-oxo-C12-HSL or 10 μM of 3-hydroxy-dodecanoyl-HSL (3-OH-C12-HSL) when appropriate, or in M9 liquid medium supplemented with 15 mM acetoin as carbon source at 23 or 30°C until an OD_600_ of 0.4–0.6 was reached, as indicated. RNA extraction and qRT-PCR were performed following procedures described in Lopez et al. (2018) and Tuttobene et al. (2018). Results are informed as normalized relative quantities (NRQs) calculated using qBASE (Hellemans et al., 2007), with *recA* and *rpoB* genes as normalizers (Muller et al., 2017). The UPL Taqman Probes (Universal Probe Library-Roche, Germany) and primers used are listed in Supplementary Table S3.

#### Growth in the Presence of Acetoin

Wild-type and derivative strains *A. baumannii* ATCC 17978 were grown on acetoin as the sole carbon source. To test the ability of the *A. baumannii* strains used in this work to grow on acetoin as the sole carbon source, 1/100 dilutions of overnight cultures grown in LB Difco were washed and inoculated in M9 liquid medium supplemented with 5, 10, or 15 mM acetoin or in LB Difco medium and grown without shaking, under blue light or in the dark at 23 or 30°C. Aliquots were removed at the times indicated in the figures in order to measure the A660 of the culture.

#### Blue Light Treatments

Blue light treatments were conducted as reported before (Mussi et al., 2010; Golic et al., 2013; Abatedaga et al., 2017; Muller et al., 2017; Tuttobene et al., 2018). Briefly, cells were grown in the dark or under blue light emitted by an array composed of 3 × 3-LED module strips emitting an intensity of 6–10 μol photons/m^2^/s, with emission peaks centered at 462 nm (Mussi et al., 2010).

#### Yeast Two-Hybrid (Y2H) Assays

Yeast two-hybrid experiments were conducted following procedures described before (Cribb and Serra, 2009; Tuttobene et al., 2018). *Saccharomyces cerevisiae* Mav 203 strain (MATa, *leu*2-3,112, *trp*1-901, *his*3-D200, *ade*2-101, *gal*4D, *gal*80D, SPAL10::URA3, GAL1::*lac*Z, HIS3UAS GAL1::HIS3, LYS2, *can*1R, and *cyh*2R) was transformed with the different expression vectors. First, BlsA and AcoN were analyzed for self-activation. For this purpose, MaV203 yeast strain containing the pGAD-T7 empty vector was transformed with the DNA DB-fusion protein expressing vectors (pGBK-X) (X = BlsA or AcoN). Conversely, MaV203 yeast strain containing the pGBK-T7 empty vector was then transformed with the AD-fusion protein expressing vectors (pGAD-Y) (Y = BlsA or AcoN). In addition, these strains were used for determination of the optimal 3-amino-1,2,4-triazole (3AT) concentration required to titrate basal *HIS3* expression. MaV203/pGBK-X strains were afterward transformed with each pGAD-Y plasmids. Transformations using one or both Y2H plasmids were performed by the lithium acetate/single-stranded carrier DNA/polyethylene glycol method described in Gietz and Woods (2002), and plated in convenient minimal selective medium [synthetic complete (SC) medium without leucine (-leu) for pGAD-Y transformants, SC without tryptophan (-trp) for pGBK-X transformants, and SC-leu-trp transformants carrying both plasmids]. The plates were then incubated at 23°C for 72 h to allow growth of transformants. A “Master Plate” was then prepared using SC-leu-trp media, in which we patched: four to six clones of each pGBK-X/pGAD-Y containing yeasts, four to six self-activation control clones pGBK-X/pGAD and pGBK/pGAD-Y (Y DNA-binding negative control), and two isolated colonies of each of the five yeast control strains (A–E). The plates were incubated for 48–72 h at 23°C. This Master Plate was then replica plated to SC–leu–trp–his+3AT and to SC–leu–trp–ura to test for growth in the absence of histidine (*his*) and uracil (*ura*), respectively (*his*3 and *ura*3 reporter activation), under the different conditions analyzed, i.e., dark/light; 23/30°C, for at least 72 h. For development of blue color as a result of β-galactosidase (β-Gal) expression, transformed yeasts were replica plated on a nitrocellulose filter on top of a YPAD medium plate and grown at the different conditions (dark/light; 23/30°C). Then, the cells on the nitrocellulose filter were permeabilized with liquid nitrogen and soaked in X-Gal solution (5-bromo-4-chloro-3-indolyl-b-D-galactopyranoside in Z buffer (60 mM Na_2_HPO_4_, 40 mM NaH_2_PO_4_, 10 mM KCl, 1 mM MgSO_4_, pH 7.0) maintaining the different incubation conditions to be tested.

### Accession Numbers

The genome of the *Acinetobacter* sp. 5-2Ac02 is deposited in GenBank database (GenBank accession number MKQS00000000; Bioproject PRJNA345289). The genome of *A. baumannii* ATCC17978 is deposited in GenBank (accession number CP018664.1). Finally, the gene expression microarray results are deposits in GEO database (GEO accession number GSE120392).

## Results

### Transcriptome Adjustments in Response to Mitomycin C Show Induction of Defense and Stress Response Systems in *Acinetobacter* sp. Strain 5-2Ac02

The airborne *Acinetobacter* sp. 5-2Ac02 isolate was first characterized to learn about its antibiotic as well as heavy metal susceptibility profiles, since its genome harbored genes of the *ter* (tellurite resistance) operon (*terZABCDEF*); *klaA* and *klaB* genes from the *kil* operon, which is associated with the previous one (O’Gara et al., 1997); as well as the arsenic-resistance operon *arsC1-arsRarsC2-ACR3-*arsH (Table 1). The data presented in Table 1 show that *Acinetobacter* sp. 5-2Ac02 is susceptible to all antibiotic tested but resistant to copper as well as to arsenic, as previously reported (Barbosa et al., 2016). This information was confirmed by growth curves in the presence of these heavy metals (Supplementary Figure S1).

Arrays performed in the presence of the stressor mitomycin C revealed induction of SOS genes such as those coding for recombinases, polymerases, as well as DNA repair proteins, all with a fold change (FC) > 3 in *Acinetobacter* sp. strain 5-2Ac02. Also, genes coding for components of six TA systems were found to be induced with a FC > 4.9 in all cases: the RelBE systems (x2), the HigBA system, the ParDE system, and two new putative systems (x2). The data also showed induction of genes involved in heavy metal resistance genes, among which can be highlighted cobalt–zinc–cadmium, copper, and arsenic resistance genes. In addition, the gene coding for colicin V protein was induced with a FC of 3.716 (Table 2). Finally, many mobile element genes, which are extraordinarily abundant in the genome of *Acinetobacter* sp. 5-2Ac02 strain, were also induced (not shown).

**TABLE 2:** Gene expression adjustments in response to mitomycin C in *Acinetobacter* sp. strain 5-2Ac02.

**Protein ID (RAST server)^a^**	**Description**	**Fold change**	**System**	**Mechanism**
202956.5.peg.1643	*recA*	5.7671	Recombinases	SOS response
202956.5.peg.129	*recT*	25.3753		
202956.5.peg.1972	*recF*	13.1351		
202956.5.peg.2285	*umuD*	5.0572	Polymerases V	
202956.5.peg.1220	*umuD*	21.6016		
202956.5.peg.2284	*umuC*	3.7093		
202956.5.peg.1221	*umuC*	4.1229		
202956.5.peg.1236	*rmlC-like cupin*	29.8694	DNA repair protein	
202956.5.peg.2036	*rmlC-like cupin*	10.1388		
202956.5.peg.274	*relB*	5.3331	RelEB system	Toxin–antitoxin modules
202956.5.peg.2563	*relB*	7.4618		
202956.5.peg.275	*relE*	5.8337		
202956.5.peg.2564	*relE*	7.8490		
202956.5.peg.411	*higA*	7.3751	HigBA system	
202956.5.peg.412	*higB*	14.9347		
202956.5.peg.2515	Antitoxin	6.7492	New putative TA system	
202956.5.peg.2516	Toxin	9.5133		
202956.5.peg.2550	Antitoxin	8.6098		
202956.5.peg.2551	Toxin	10.5546		
202956.5.peg.797	*parD*	5.8254	ParDE system	
202956.5.peg.796	*parE*	4.9563		
202956.5.peg.984	*aphC*	2.3370	Reductase	Oxidant tolerance (ROS response)
202956.5.peg.319	*rpoS* regulon	28.4676	Regulatory system	
202956.5.peg.1019	*arsC* (arsenate reductase)	3.7665	Reductase	Heavy metals resistance
202956.5.peg.1000	*copA* (*copD*)	2.1898	Copper resistance operon	
202956.5.peg.1009	*copB*	2.3034		
202956.5.peg.1001	*copC*	3.2015		
202956.5.peg.2458	*czcA*	4.6191	Cobalt–zinc–cadmium resistance operon	
202956.5.peg.1476	*czcD*	3.1468		
202956.5.peg.2744	*cvpA* (colicin V)	3.7163	Bacteriocin protein	Antibiotic peptides

*Genes showing FC >2 are indicated. ${}^{a}$RAST server was used to identify the protein-coding genes, rRNA and tRNA genes, and to assign predictive functions to these genes.*

**FIGURE 1:**
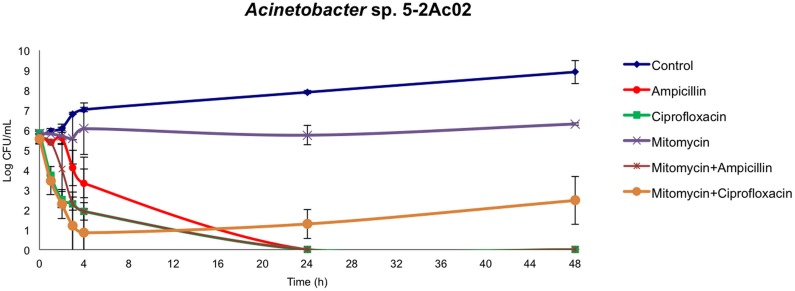
Killing curves of *Acinetobacter* sp. 5-2Ac02 in the presence of antimicrobials and/or mitomycin C.

The TA systems have been shown to be involved both in tolerance and persistence (Fernandez-Garcia et al., 2018). We next analyzed the fraction of tolerant or persister cells in populations of *Acinetobacter* sp. strain 5-2Ac02 by determining the time-kill responses in the presence of ampicillin, ciprofloxacin, mitomycin C, and combinations of these (Figure 1), following protocols described in Hofsteenge et al. (2013). The data show a large decrease in colonies of *Acinetobacter* sp. strain 5-2Ac02 during the first 24 h in the presence of ampicillin, ciprofloxacin, as well as in the presence of the combination of ampicillin and mitomycin C. Interestingly, the presence of a combination of mitomycin C with ciprofloxacin showed a tolerant population displaying slow growth at 4, 24, and 48 h (Figure 1) under this stress condition, which may result from activation of defense mechanisms such as the toxins and antitoxins systems as well as SOS response.

### Quorum Sensing Signals Modulate Expression of the Acetoin/Butanediol Catabolic Cluster in *Acinetobacter* spp., Being AcoN a Negative Regulator in *A. baumannii*

**FIGURE 2:**
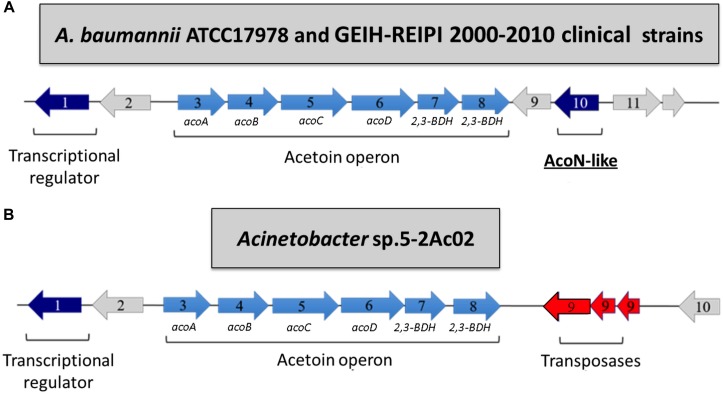
Acetoin/butanediol cluster from *A. baumannii* ATCC 17978 (GenBank accession number CP018664.1; *acoN*-like AU097_16290) as well as clinical *A. baumannii* strains isolated in the “II Spanish Study of *A. baumannii* GEIH-REIPI 2000–2010” involving 45 Spanish hospitals with 246 patients (GenBank Umbrella Bioproject PRJNA422585) **(A)** and Acinetobacter sp. strain 5-2Ac02 (GenBank accession number MKQS00000000; Bioproject PRJNA345289) **(B)**. The following proteins from the acetoin/butanediol cluster are indicated by arrows: (1) putative transcriptional regulator; (2) hypothetical protein; (3) *acoA*, acetoin dehydrogenase E1 alpha-subunit; (4) *acoB*, acetoin dehydrogenase E1 beta-subunit; (5) *acoC*, dihydrolipoamide acetyltransferase (E2) acetoin; (6) *acoD*, dihydrolipoamide dehydrogenase subunit of acetoin dehydrogenase; (7) 2,3-BDH/2,3-butanediol dehydrogenase, S-alcohol forming, (S)-acetoin-specific; (8) 2,3-BDH/2,3-butanediol dehydrogenase, R-alcohol forming, (R)- and (S)-acetoin-specific; (9) hypothetical protein (*A. baumannii* ATCC17978) and transposases (*Acinetobacter* sp. 5-2Ac02 strain); (10) putative transcriptional regulator (AcoN, *A. baumannii* ATCC17978) and hypothetical protein (*Acinetobacter* sp. 5-2Ac02 strain).

Array expression studies of *Acinetobacter* sp. 5-2Ac02 in the presence of a mixture of *N*-acyl-homoserine lactones (AHLs) or 3-oxo-C12-HSL, which are modulators of the quorum network in *A. baumannii* (Lopez et al., 2018), indicated induction of the acetoin/butanediol catabolic pathway genes, each with a FC > 1.5 (Tables 3, 4, respectively). We show the genomic arrangement of this cluster in the genomes of *Acinetobacter* sp. 5-2Ac02 and *A. baumannii* ATCC 17978 strain (Figures 2A,B). The same genomic configuration in *A. baumannii* strain ATCC 17978 was observed in 18 clinical *A. baumannii* strains isolated in the “II Spanish Study of *A. baumannii* GEIH-REIPI 2000–2010” which included 45 Spanish hospitals with 246 patients (GenBank Umbrella Bioproject PRJNA422585) (Supplementary Table S4).

**TABLE 3:** Expression of genes in *Acinetobacter* sp. strain 5-2Ac02 by quorum network molecules (AHLs mix).

**Protein ID (RAST server)^a^**	**Gene/predicted protein description**	**Fold change**	**System**	**Mechanism**
202956.5.peg.1419	*acoA*/acetoin dehydrogenase E1 alpha-subunit	3.9966	Acetoin/butanediol cluster (degradation)	QS system
202956.5.peg.1420	*acoB*/acetoin dehydrogenase E1 beta-subunit	3.7291		
202956.5.peg.1421	*acoC*/dihydrolipoamide acetyltransferase (E2) acetoin	3.6752		
202956.5.peg.1422	*acoD*/dihydrolipoamide dehydrogenase of acetoin dehydrogenase	3.3919		
202956.5.peg.1423	2,3-BDH/2,3-butanediol dehydrogenase, S-alcohol forming, (S)-acetoin-specific	2.9770		
202956.5.peg.1424	2,3-BDH/2,3-butanediol dehydrogenase, R-alcohol forming, (R)- and (S)-acetoin-specific	1.9456		
202956.5.peg.2091	1,2-Dihydroxycyclohexa-3,5-diene-1-carboxylate dehydrogenase	2.4396	Aromatic compounds biodegradation cluster	QS system
202956.5.peg.2092	Benzoate dioxygenase, ferredoxin reductase	2.8198		
202956.5.peg.2093	Benzoate 1,2-dioxygenase beta-subunit	3.2909		
202956.5.peg.2094	Benzoate 1,2-dioxygenase alpha-subunit	3.3731		
202956.5.peg.2095	Catechol 1,2-dioxygenase	3.5774		
202956.5.peg.2096	Muconolactone isomerase	3.1273		

*${}^{a}$RAST server was used to identify the protein-coding genes, rRNA and tRNA genes, and to assign functions to these genes.*

**TABLE 4:** Expression of genes in *Acinetobacter* sp. strain 5-2Ac02 by quorum network molecules (3-oxo-C12-HSL).

**Protein ID (RAST server)${}^{\text{a}}$**	**Gene/predicted protein description**	**Fold change**	**System**	**Mechanism**
202956.5.peg.1419	*acoA*/acetoin dehydrogenase E1 alpha-subunit	2.3803	Acetoin/butanediol cluster	QS system
202956.5.peg.1420	*acoB*/acetoin dehydrogenase E1 beta-subunit	2.5212		
202956.5.peg.1421	*acoC*/dihydrolipoamide acetyltransferase (E2) of acetoin dehydrogenase complex	2.7127	Acetoin/butanediol cluster	QS system
202956.5.peg.1422	*acoD*/dihydrolipoamide dehydrogenase of acetoin dehydrogenase	2.2546		
202956.5.peg.1423	2,3-BDH/2,3-butanediol dehydrogenase, S-alcohol forming, (S)-acetoin-specific	2.0217		
202956.5.peg.2196	Aminoacid transporter	3.6649		Others
202956.5.peg.2630	Short-chain dehydrogenase	2.8179		
202956.5.peg.2505	Amide	2.6736		
202956.5.peg.2388	Transporter (DMT) superfamily	2.6240		
202956.5.peg.2137	Alcohol dehydrogenase	2.5237		
202956.5.peg.517	Ribonucleotide reductase	2.3017		
202956.5.peg.2100	dcaP	2.2854		
202956.5.peg.1418	Lipoate synthase	2.2626		
202956.5.peg.691	Monooxygenase	2.1241		
202956.5.peg.2775	Cyclic AMP receptor	2.0731		
202956.5.peg.2753	*cspA*	2.0635		
202956.5.peg.451	NAD(P)	2.0352		
202956.5.peg.434	Protein-export membrane protein SecD	2.0152		
202956.5.peg.1121	Putrescine importer	2.0026		

*^a^RAST server was used to identify the protein-coding genes, rRNA and tRNA genes, and to assign functions to these genes.*

Ten genes were identified in the ATCC 17978 cluster, likely coding for a putative transcriptional regulator (gene 1) followed by a putative lipoyl synthase (gene 2), two oxidoreductases homologous to *acoA* and *acoB* (genes 3 and 4), a deaminase homologous to *acoC* (gene 5), a dehydrogenase homologous to *acoD* (gene 6), a BDH reductase (gene 7), and a BDH (gene 8), all of which are followed by a hypothetical protein (gene 9) and a putative transcriptional regulator (gene 10) (Figure 2A). Gene 2 is homologous to *acoK* (Figure 2A) and gene 1 is homologous to a positive transcriptional regulator (activator) homologous to *acoR* in different organisms (Figure 2A). The genomic configuration in *Acinetobacter* sp. strain 5-2Ac02 is similar to that of ATCC 17978 except that genes coding for the hypothetical protein and the putative transcriptional regulator (9 and 10 in ATCC 17978, respectively) are absent, while three genes coding for putative transposases were identified following gene 8 (Figure 2B).

Finally, in presence of the AHL mixture, the arrays also revealed increased expression (FC > 2) of genes involved in biodegradation of aromatic compounds (Table 4).

We suspected that the absence of the putative transcriptional regulator in *Acinetobacter* sp. strain 5-2Ac02, designated as gene 10 in the genome locus of *A. baumannii* ATCC17978 (Figure 2A) and renamed here from now on as *acoN*, might be responsible for the induced expression of the acetoin catabolic genes in response to quorum network signals.

**FIGURE 3:**
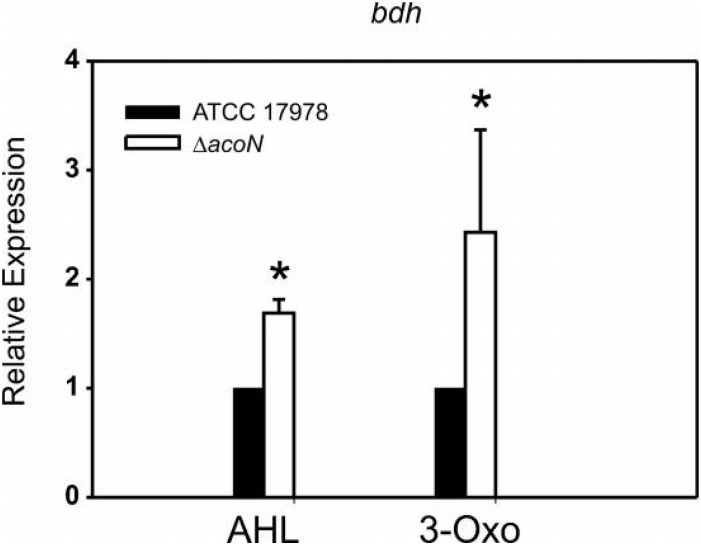
The BDH (*bdh*) gene is induced by quorum network signals in the *ΔacoN* mutant. Estimation of the relative levels of the BDH mRNA by qRT-PCR in the presence of AHLs or 3-oxo-C12-HSL in the wild-type *A. baumannii* ATCC 17978 and *ΔacoN* genetic backgrounds. The data shown are mean ± SD of the expression levels relative to the wild type from at least three biological replicates. Asterisks indicate significant differences in *ΔacoN* compared to wild type as indicated by *t*-test (*p* < 0.01).

We reasoned that whether this was the case, then a knockout mutant in *acoN* in *A. baumannii* ATCC 17978, which would resemble the situation in the so far genetically intractable *Acinetobacter* sp. strain 5-2Ac02, would result in induction of the acetoin catabolic genes in the presence of quorum sensing signals. As can be observed in Figure 3, the presence of quorum sensing signals resulted in induction of the transcript levels of BDH (*bdh*, acetoin/butanediol cluster) (RE > twofold) in the *A. baumannii* ATCC 17978 *ΔacoN* mutant with respect to the wild-type strain. This provides the first clue that AcoN functions as a negative regulator of acetoin catabolic genes.

**FIGURE 4:**
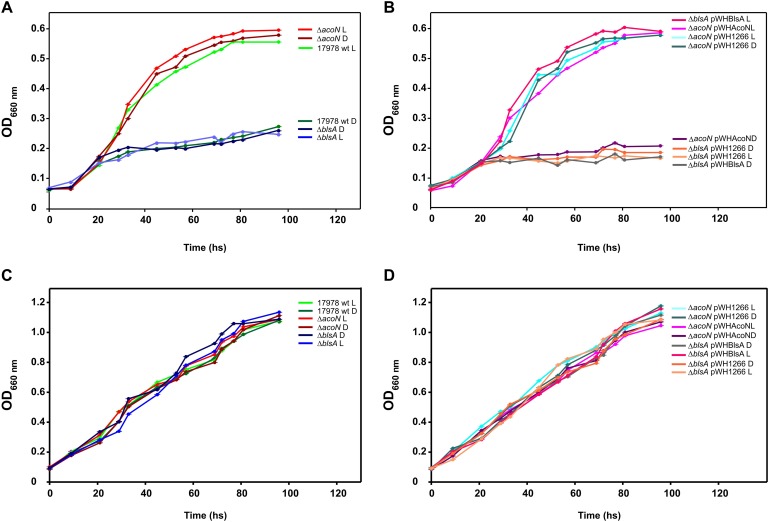
Light modulates acetoin catabolism at moderate temperatures in *A. baumannii* ATCC 17978. **(A,B)** Growth curves in M9 minimal medium supplemented with acetoin 5 mM as sole carbon source of *A. baumannii* ATCC 17978 wild-type and derivative strains, incubated stagnantly at 23°C under blue light (L) or in the dark (D). **(C,D)** Growth curves in LB medium of *A. baumannii* ATCC 17978 wild-type and derivative strains incubated stagnantly at 23°C under blue light (L) or in the dark (D). Growth was measured by determining the optical density at 660 nm. The experiments were performed at least in triplicate, including three replicates for each strain at each condition. Representative results are shown.

Further studies showed that the *ΔacoN* mutant grew much better in media supplemented with acetoin (5 mM) as sole carbon source than the wild-type strain in the dark at 23°C (Figure 4A), which barely grew at this condition. The *ΔacoN* mutant containing the pWHAcoN plasmid, which expresses *acoN* directed from its own promoter, behaved as the wild type showing a reduced ability to grow on acetoin as sole carbon source at 23°C in the dark, restoring therefore the wild-type phenotype (Figure 4B). Similar results were obtained at 30°C and are discussed later in the manuscript. These results provide further evidence of the role of AcoN gene as a negative regulator of the acetoin catabolic cluster.

**FIGURE 5:**
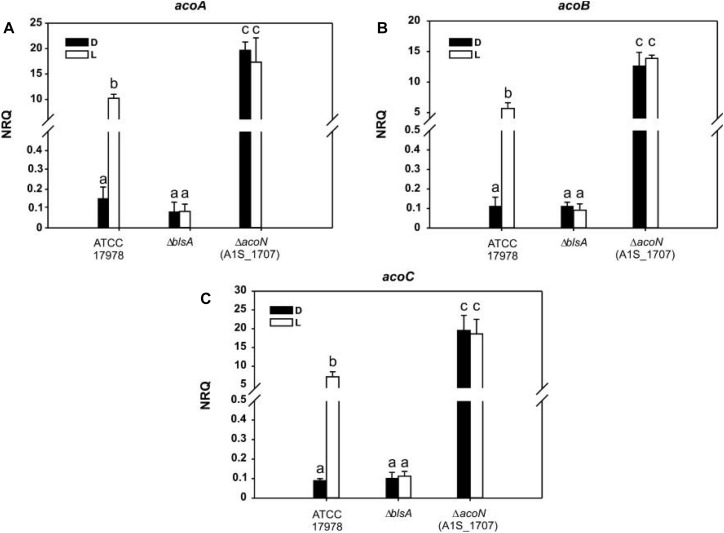
Acetoin catabolism genes are induced under blue light at moderate temperatures. **(A–C)** Estimation of the expression levels of representative genes of the acetoin catabolic cluster, acoA–C, by RT-qPCR in *A. baumannii* ATCC 17978 wild-type as well as *ΔblsA* and *ΔacoN* genetic backgrounds at 23°C under blue light (L) or in the dark (D). The data shown are mean ± SD of normalized relative quantities (NRQs) calculated from transcript levels measured in samples grown in M9 minimal media supplemented with acetoin as sole carbon source under blue light or in the dark at 23°C, in at least three biological replicates. Different letters indicate significant differences as determined by ANOVA followed by Tukey’s multiple comparison test (*p* < 0.01).

Finally, expression of acetoin catabolic genes such as *acoA*, *acoB*, and *acoC* was induced approximately 150-folds in the *ΔacoN* mutant with respect to the wild type at 23°C in the dark (Figure 5). These results confirm the functioning of AcoN as a negative regulator of the acetoin catabolic pathway in *A. baumannii*.

### Light Modulates Acetoin Catabolism Through BlsA and AcoN at Moderate Temperatures in *A. baumannii*

Acetoin catabolic genes such as *acoA*, *acoB*, *acoC*, and *acoD* have been previously shown to be induced by light at moderate temperatures in *A. baumannii* ATCC 19606 by RNA-seq studies (Muller et al., 2017). We thus studied whether light modulated acetoin catabolism in ATCC 17978 at 23°C and found a differential ability of this strain to grow in the presence of acetoin as sole carbon source between light and dark conditions (Figure 4 and Supplementary Figure S2).

Figure 4A shows that *A. baumannii* ATCC 17978 grows much poorer in 5 mM acetoin in the dark rather than under blue light at 23°C. The *ΔblsA* mutant, which lacks the only traditional photoreceptor encoded in the *A. baumannii* genome, behaved as the wild type in the dark both under blue light or in the dark (Figure 4A), as also did the mutant containing the empty vector pWH1266 (Figure 4B). In contrast, the *ΔblsA* mutant containing pWHBlsA, which expresses *blsA* directed from its own promoter, grew better on acetoin under blue light than in the dark, restoring thus the wild-type phenotype (Figure 4B). The *ΔacoN* mutant, both under blue light and in the dark, behaved as the wild type under blue light, i.e., showed enhanced growth with respect to the wild type in the dark, congruent with the absence of the negative regulator (Figure 4A); as also did the *ΔacoN* mutant containing pWH1266 (Figure 4B). The *ΔacoN* mutant containing pWHAcoN, which expresses *acoN* directed from its own promoter, grew better on acetoin under blue light than in the dark, therefore restoring the wild-type phenotype (Figure 4B). Similar results were obtained when acetoin 10 and 15 mM was used as sole carbon source (Supplementary Figure S2).

These results show that light modulation of acetoin catabolism depends on the BlsA photoreceptor and the AcoN negative regulator in *A. baumannii* ATCC 17978. Opposite behavior is observed for *ΔblsA* and *ΔacoN* mutants regarding modulation of growth on acetoin by light, indicating that BlsA is necessary for the observed induction, while AcoN for repression. The overall evidence prompts us to postulate a model in which BlsA interacts with AcoN under blue light at 23°C antagonizing this repressor, with the concomitant induction of acetoin catabolic genes’ expression as well as growth on acetoin in this condition. It is important to mention that the viability of cells was not affected by light, as similar growth curves were obtained for the different strains in the complex media LB under blue light and in the dark (Figures 4C,D).

### Light Regulates Expression of the Acetoin Catabolic Pathway Through BlsA and AcoN at Moderate Temperatures in *A. baumannii*

We then monitored AcoN functioning in response to light by measuring the expression of AcoN-regulated genes under different illumination conditions and genetic backgrounds. To this end, the expression of the acetoin catabolic genes *acoA*, *acoB*, and *acoC* (Figures 5A–C respectively) was analyzed by qRT-PCR at different light conditions at moderate temperatures in *A. baumannii* strain ATCC 17978. Our results show that the expression levels of these genes were basal in the dark at 23°C in M9 minimal medium with acetoin as sole carbon source. However, their expression was significantly induced in the presence of blue light (Figure 5). In *ΔblsA* mutants, expression of *acoA–C* genes was basal and comparable between blue light and dark, and similar to that observed for the wild type in the dark at 23°C (Figure 5). Thus, light modulates the expression of the acetoin catabolic genes, *acoA–C* through BlsA. On its side, the *ΔacoN* mutant also lost photoregulation, i.e., expression levels of *acoA–C* genes were similar between the illuminated or dark conditions. However, for this mutant, expression levels were much higher even than those registered in the wild-type under blue light, i.e., in the induced condition (Figure 5). Indeed, *acoA* expression levels in the *ΔacoN* mutant were approximately twofold higher than in the wild type under blue light, while *acoB* and *acoC* expression levels were about threefold higher, and >100-folds higher than the wild type in the dark. Opposite behavior is observed for *ΔblsA* and *ΔacoN* mutants regarding modulation of *acoA–C* genes’ expression, suggesting that BlsA is necessary for the observed induction while AcoN for repression. Altogether, BlsA antagonizes the functioning of AcoN under blue light at 23°C, with the concomitant induction of the expression of AcoN-regulated genes at this condition. By analogy with a mechanism described previously for BlsA and Fur (Tuttobene et al., 2018), we hypothesized that BlsA might interact with the AcoN negative regulator, antagonizing its functioning.

### BlsA Interacts With the Acetoin Catabolic Negative Regulator AcoN Under Illumination at Moderate Temperatures in *A. baumannii*

**FIGURE 6:**
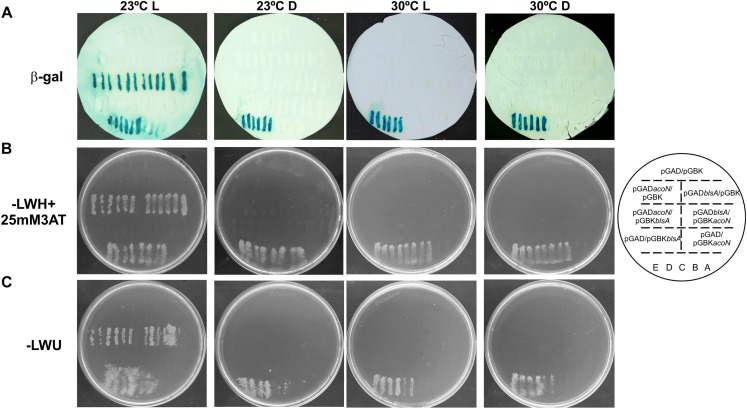
BlsA interacts with AcoN only under blue light at moderate temperatures in *A. baumannii*. BlsA–AcoN interaction was analyzed by Y2H assays at different conditions including 23°C under blue light (L) or in the dark (D), and 30°C under blue light (L) or in the dark, following procedures described in Cribb and Serra (2009) and Tuttobene et al. (2018). In each plate were patched six clones of MaV203/pGAD-*blsA* or MaV203/pGAD-*acoN* transformed with plasmids pGBK-*acoN* or pGBK-*blsA*, respectively, as well as plasmid pGBK-T7 as negative control. Reciprocal combinations were also included, as well as self-activation and different strength interaction controls (strains A–E). The scheme on the right side represents the order of yeast streaks on each plate. Panel **A** shows results for the *lacZ* reporter, panel **B** for the histidine auxothropic reporter and panel **C** for the uracil reporter. Experiments were performed in triplicates and representative results are shown.

Yeast two-hybrid assay experiments were conducted to study if BlsA interacts with AcoN, using an adapted system from ProQuest^TM^ Two-Hybrid System, as previously described (Tuttobene et al., 2018). The system includes strain Mav 203, which harbors three reporter genes with different promoters to avoid false positives: *lacZ* and two auxotrophic markers HIS3 and URA3. If the two proteins studied do interact, the appearance of blue color as well as growth in the absence of histidine or uracil would be observed. Gateway-system vectors pGAD-T7Gw and pGBK-T7Gw adapted to Y2H express each of the studied genes, *blsA* and *acoN*, as fusions to GAL4 DNA DB or AD. In each plate were also included self-activation controls (pGAD-T7Gw and pGBK-T7Gw empty vectors) as well as different strength interaction controls (A–E), to give an indication of the reporter genes’ expression levels. In our previous report (Tuttobene et al., 2018), we observed that BlsA protein interactions depend on illumination and temperature conditions, so we decided to test its interaction with AcoN, the acetoin catabolism negative regulator, under different conditions. Figure 6 shows results of Y2H assay experiments at the different conditions analyzed. At 23°C under blue light (Figure 6), the interaction between BlsA and AcoN was demonstrated by the appearance of blue color and growth in SC defined media without the supplementation of histidine or uracil, i.e., results were consistent for the three reporters analyzed. The interactions occurred independently of the vector used, as both pGAD*blsA*/pGBK*acoN* and pGAD*acoN*/pGBK*blsA* combinations produced signals (Table 5). Growth on SC–Ura plates indicates a strong interaction between BlsA and AcoN in the conditions analyzed, since the *URA3* reporter is the least sensitive^1^. Moreover, controls indicated absence of self-activation of each protein fused to DB or AD: (pGAD-T7/pGBK*blsA* or pGBK*acoN*) or (pGBK-T7/pGAD*blsA* or pGAD*acoN*) (Table 5). The overall data provide convincing evidence indicating that BlsA interacts with AcoN at 23°C under blue light. However, no positive signal was detected for AcoN–BlsA interaction by Y2H assays for any of the reporters tested at 23°C in the dark, while interaction controls behaved as expected (Figure 6 and Table 5). Altogether, the data account for BlsA interacting with AcoN in a light-dependent manner at moderate temperatures. Table 5 summarizes the results obtained for Y2H.

**TABLE 5:** The interaction between AcoN and BlsA was determined by the yeast two hybrid assay, using GAL4 activation domain (AD) and DNA-binding domain (BD) fusion proteins.

**β-Gal**	**Empty vector pGAD-T7**	**BlsA_AD**	**AcoN_AD**
Empty vector pGBK-T7	−	−	−
AcoN BD	−	**+**	ND
BlsA_BD	−	ND	**+**
***HIS 3***			
Empty vector pGBK-T7	−	−	−
AcoN BD	−	**+**	ND
BlsA_BD	−	ND	**+**
***URA3***			
Empty vector pGBK-T7	−	−	−
AcoN BD	−	**+**	ND
BlsA_BD	−	ND	**+**

*Both combinations of fusion proteins (AcoN_BD vs. BlsA_AD and BlsA _BD vs. AcoN_AD) were assayed giving the same results with all three reporter genes (β-Gal, HIS 3, and URA 3). +, means reporter gene expression induced by a positive interaction; −, means no interaction, confirming that no “self-activation” of the fusion proteins may result in the reporters expression (interactions using pGAD-T7 and pGBK-T7 empty vectors in combination with the fusion proteins); ND, means that self-interactions of AcoN and BlsA were not determined.*

### AcoN Does Not Modulate A1S_1697 Expression in Response to Light

**FIGURE 7:**
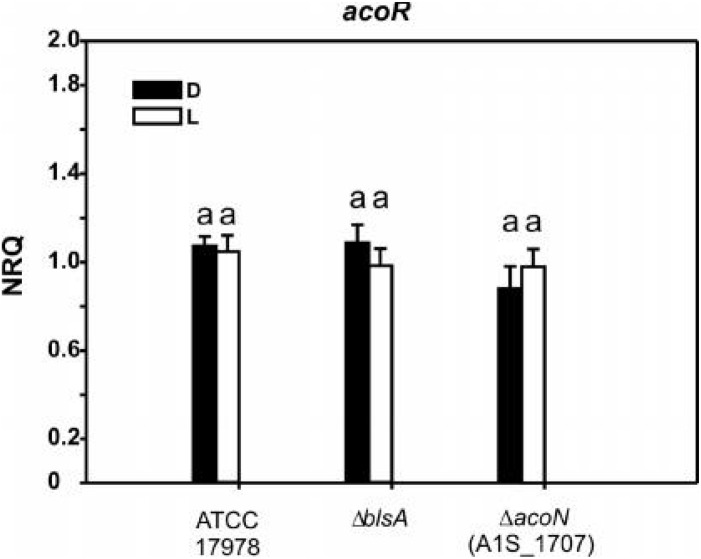
A1S_1697 expression does not depend on light nor on AcoN. Estimation of the expression levels of A1S_1697 by RT-qPCR in *A. baumannii* ATCC 17978 wild-type as well as *ΔblsA* and *ΔacoN* genetic backgrounds at 23°C under blue light (L) or in the dark (D). The data shown are mean ± SD of NRQs calculated from transcript levels measured in samples grown in M9 minimal media supplemented with acetoin as sole carbon source under blue light or in the dark at 23°C, in at least three biological replicates. Different letters indicate significant differences as determined by ANOVA followed by Tukey’s multiple comparison test (*p* < 0.01).

We next analyzed the possibility that AcoN would be directly controlling the expression of the other putative transcriptional regulator identified in this cluster (gene 1, A1S_1697) in *A. baumannii* (Figure 2), which by analogy with *acoR* from *B. subtilis* might be an activator of the acetoin cluster. Whether this hypothesis is correct, AcoN would modulate *acoA–C* in response to light indirectly by modulation of the functioning of the putative activator. For this purpose, we studied A1S_1697 expression at different illumination conditions and genetic backgrounds. If AcoN functions as a negative regulator of A1S_1697 expression in a light-dependent manner, then A1S_1697 transcripts levels would vary between light and dark conditions. This variation would level in the *ΔacoN* mutant between light and dark, and reach higher expression levels than the wild type, had it been the negative regulator. However, and as seen in Figure 7, A1S_1697 transcripts levels were similar between light and dark for all the genetic backgrounds analyzed, namely, the wild-type strain, and the *ΔblsA* and *ΔacoN* mutants. These results indicate that AcoN does not regulate A1S_1697 expression in response to light.

### BlsA–AcoN Interaction Is Significantly Reduced at Higher Temperatures

Since BlsA and AcoN interact at 23°C under blue light, we wondered whether this interaction is conserved at higher temperatures. Thus, BlsA–AcoN interactions were studied by Y2H at a temperature that supports yeast growth such as 30°C. A control at 23°C under blue light was always included for each repetition. Figure 6 shows representative Y2H results indicating null or negligible BlsA–AcoN interactions at 30°C, neither in the dark nor under blue light.

### Light Does Not Modulate Acetoin Catabolism at Higher Temperatures

**FIGURE 8:**
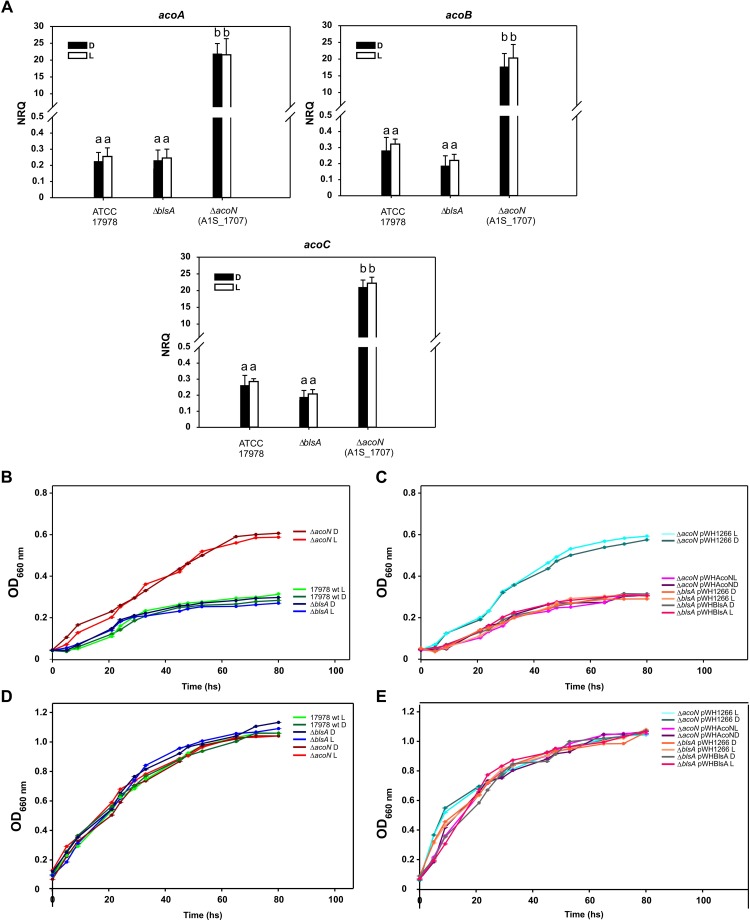
Light does not modulate acetoin catabolism at higher temperatures. **(A)** Estimation of the expression levels of representative genes components of the acetoin catabolic cluster, *acoA–C*, by RT-qPCR in *A. baumannii* ATCC 17978 wild-type as well as *ΔblsA* and *ΔacoN* genetic backgrounds under blue light (L) or in the dark (D) at 30°C. The data shown are mean ± SD of NRQs calculated from transcript levels measured in samples grown in M9 minimal media supplemented with acetoin as sole carbon source under blue light or in the dark at 30°C, in at least three biological replicates. Different letters indicate significant differences as determined by ANOVA followed by Tukey’s multiple comparison test (*p* < 0.01). **(B,C)** Growth curves in M9 minimal media supplemented with acetoin as sole carbon source of *A. baumannii* ATCC 17978 wild-type and derivative strains incubated stagnantly under blue light or in the dark at 30°C. **(D,E)** Growth curves of *A. baumannii* ATCC 17978 wild-type and derivative strains in LB media incubated stagnantly under blue light (L) or in the dark (D) at 30°C. Growth was measured by determining the optical density at 660 nm. The experiments were performed at least in triplicates, including three repetitions for each strain at each condition. Representative results are shown.

We next studied whether acetoin catabolic gene expression and growth was modulated by light at 30°C, since no interaction between BlsA and AcoN was detected at this temperature. As expected, *acoA*, *acoB*, and *acoC* gene expression showed no differential modulation by light neither in *A. baumannii* ATCC 17978 wild type, nor in the *ΔblsA* or *ΔacoN* mutants at this condition (Figure 8A). At 30°C, *acoA–C* expression levels in the *ΔblsA* mutant were similar to the wild-type strain both under blue light and in the dark, i.e., were repressed; while they were induced in the *ΔacoN* mutant both under blue light and in the dark. This behavior was congruent with growth curves performed in M9 minimal media supplemented with acetoin as sole carbon source, which showed no significant difference between light and dark for any of the studied strains (Figures 8B,C). Here again, the *ΔacoN* mutant showed enhanced growth consistent with the absence of the negative regulator, as also did the *ΔacoN* mutant containing pWH1266 (Figures 8B,C). The overall data indicate that light does not influence acetoin catabolism at 30°C or above, and are in agreement with available knowledge regarding BlsA functioning (Mussi et al., 2010; Golic et al., 2013; Abatedaga et al., 2017).

## Discussion

*Acinetobacter* sp. are extremely well adapted to different hostile environments thanks to several molecular mechanisms that enable survival under stress conditions. Here, we characterized the *Acinetobacter* sp. 5-2Ac02 strain isolated from the air in a hospital from Brazil. *Acinetobacter* sp. 5-2Ac02 showed an antibiotic susceptible profile. It includes a *bla*_oxa–58_ gene as well as *tet* genes, which have been related to resistance to tetracycline, coded in its genome. This susceptible strain carrying these cryptic genes hence represents a clinical threat as it may act as a reservoir of resistance genes. The high arsenic MIC for *Acinetobacter* sp. strain 5-2Ac02 may be attributed to the arsenic operon, arsC1–arsR–arsC2–ACR–arsH, which has only been described in the *Pseudomonas stutzeri* TS44 (Barbosa et al., 2016).

We further analyzed the global gene expression adjustments in this strain in response to environmental stressors such as mitomycin C and found induction of genes coding for components of the SOS response, genes involved in numerous TA systems (RelBE, HigBA, parDE, and other two new TA systems) (Barbosa et al., 2016), and resistance to heavy metals and antioxidant enzymes. The TA systems have been shown to be involved both in tolerance and persistence, which presuppose the ability of the bacteria to grow slowly or enter into a dormant state, respectively, to cope with the presence of a stressor (Fernandez-Garcia et al., 2018). It is thus not surprising that in the presence of mitomycin C and ciprofloxacin a tolerance phenotype was observed in killing curves (Figure 1). Furthermore, the ability of *A. baumannii* to survive for long periods of desiccation has been related to the achievement of dormant states, via mechanisms affecting control of cell cycling, DNA coiling, transcriptional and translational regulation, protein stabilization, antimicrobial resistance, and toxin synthesis (Gayoso et al., 2014). The fact that this airborne strain, in which desiccation is a common feature in its lifestyle, harbors and modulates numerous determinants leading to persistence in adverse environmental conditions is thus aligned with this notion.

Under pressure from the quorum network, both AHLs and 3-oxo-C12-HSL compounds induced the expression of a cluster involved in acetoin/butanediol metabolism in *Acinetobacter* sp. 5-2Ac02, which was also shown to be induced by light in *A. baumannii* (Muller et al., 2017). Acetoin (3-hydroxy-2-butanone) is a four carbon neutral molecule used as substrate by various microorganisms, with multiple usages in flavor, cosmetic, and chemical synthesis (Xiao et al., 2007). In *B. subtilis*, acetoin is a significant product generated from glucose metabolism in aerobiosis. Given its neutral nature, acetoin allows the consumption of important quantities of glucose without acidification of the medium. It can also serve as a carbon reserve which can be expelled to the exterior and later re-internalized (Ali et al., 2001). Acetoin and BD are also BVCs, which can influence bacterial pathogenesis (Audrain et al., 2015) by altering the production of virulence factors (Venkataraman et al., 2014) or by affecting host cell functions (Kurita-Ochiai et al., 1995). In addition to the fundamental ecological interest, a better understanding of environmental bacteria and of the roles of BVCs (including BD), metabolic pathways, and mechanisms involved could provide new information about the bacterial response to the environment, thus potentially leading to clinical or industrial applications.

**FIGURE 9:**
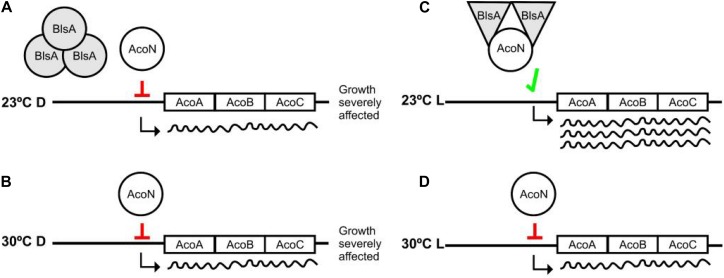
Working model representing photoregulation of acetoin catabolism through AcoN and BlsA. At 23°C in the dark, BlsA and AcoN do not interact, and AcoN represses expression of the acetoin catabolic genes acoA, acoB, and acoC **(A)**. As a result, growth on acetoin as sole carbon source is severely affected. Under blue light, BlsA acquires an excited state now capable of interacting with AcoN, antagonizing its functioning, allowing expression from the acetoin catabolic operon, and supporting growth **(B)**. Overall, BlsA finely tunes AcoN levels in response to light, modulating therefore acetoin catabolism. At 30°C, both under blue light or in the dark, BlsA does not interact with AcoN maintaining therefore its functioning as a repressor **(C,D)**, resulting growth severely affected at this condition.

Comparisons of the genetic organization of this cluster from *Acinetobacter* sp. 5-2Ac02 with that of *A. baumannii* ATCC 17978 guided us to further study a gene annotated as a putative transcriptional regulator, then designated AcoN by us. We show here that it behaves as a negative regulator of the acetoin/butanediol cluster in an *A. baumannii* and is involved in photoregulation of acetoin catabolism in *A. baumannii* through the photoreceptor BlsA. In this context, we have recently shown that BlsA binds to and antagonizes the functioning of the transcriptional repressor Fur only in the dark at 23°C, likely by reducing its ability to bind to acinetobactin promoters with the concomitant enhanced gene expression and growth under iron deprivation at this condition (Tuttobene et al., 2018). In this work, we have broadened our understanding of BlsA functioning by showing that this photoreceptor can antagonize the functioning of other transcriptional regulators also under blue light such as AcoN. Our results support a model in which the system is at a basal level or repressed state in most conditions, for example in the dark at 23°C as well as at 30°C both in the dark or under blue light, i.e., AcoN is repressing acetoin catabolic genes’ transcription resulting in basal gene expression levels as well as severely affected growth on acetoin (Figure 9). However, under blue light at 23°C the system gets derepressed: BlsA binds to the acetoin repressor AcoN antagonizing its functioning, likely by reducing its ability to bind to acetoin catabolic genes’ promoters, allowing thus their expression at this condition (Figure 9). Overall, the global regulator BlsA functions both under blue light and in the dark at low–moderate temperatures modulating different transcriptional regulators, such as Fur and AcoN, as well as the corresponding sets of regulated genes and the corresponding cellular processes. In this sense, BlsA probes to be unique among described photoreceptors regarding its dual activity under illumination and in the dark. Indeed, many photoreceptors have been shown to antagonize transcriptional repressors (Tuttobene et al., 2018), such as AppA from *Rhodobacter sphaeroides* (Pandey et al., 2017), PixD from *Synechocystis* sp. PCC6803 (Fujisawa and Masuda, 2017), and YcgF from *E. coli* (Tschowri et al., 2012). However, their functioning has been reported to occur in the dark for the first two or under blue light for the last one. This constitutes therefore the first report showing that a single photoreceptor can act both under blue light and in the dark for differential modulation by light of diverse cellular processes.

The fact that BlsA-Fur modulates photoregulation of iron uptake, while BlsA–AcoN modulates photoregulation of acetoin catabolism in *A. baumannii* at low–moderate temperatures such as 23°C but not 30°C, is consistent with previous findings of our group. In fact, we have previously showed that BlsA integrates a temperature signal in addition to light by mechanisms affecting different points of regulation. On the one side, *blsA* expression levels are very much reduced at 30 or 37°C with respect to 23°C, which correlates with negligible photoreceptor levels in the cells at 37°C (Abatedaga et al., 2017; Tuttobene et al., 2018), while the other point of control by temperature affects BlsA photoactivity (Abatedaga et al., 2017).

The mechanism by which BlsA perceives light and differentially binds to transcriptional regulators is not clear and could result from differential properties displayed by the photoreceptor at each condition, for example regarding the oligomerization state. In this sense, our results show that BlsA forms oligomers both under blue light or in the dark at 23°C (Tuttobene et al., 2018). Yet, variations in the composition or order level of these oligomers at each condition could account for differential functioning, as is the case of *Synechocystis* sp. PCC6803 PixD (Fujisawa and Masuda, 2017).

Many questions arise from our findings such as why photoregulation of acetoin catabolism at moderate temperatures has evolved in this pathogen. Likely, the answer lies in the lifestyle carried out by the microorganism at this condition. In this context, and as mentioned before, it has been shown that utilization of BD, a common fermentation product of *P. aeruginosa* co-habitant bacteria, significantly increases virulence and infection of the microorganism (Venkataraman et al., 2014; Nguyen et al., 2016; Liu et al., 2018). The activation of the pathway of BD utilization through acetoin by light observed could plausibly go in this same sense too in *A. baumannii*. Indeed, we have already seen that light induces factors related to virulence and/or persistence in the environment such as the type VI secretion system T6SS, the phenylacetic acid catabolic pathway, trehalose biosynthesis, tolerance to antibiotics, production of antioxidant enzymes, etc. (Muller et al., 2017), which could ultimately contribute to persistence and competition with other microorganisms in the habitat.

Future experiments will be devoted to provide a detailed characterization of the mechanism of photoregulation directed by BlsA, AcoN, and their targets. First, we will conduct gel mobility assays (EMSA) to prove that AcoN is a DNA-binding transcriptional regulator, as is strongly suggested by BLAST sequence homology analyses, which show 97–100% identity with proteins annotated as sigma-54-dependent Fis family DNA-binding transcriptional regulators in *A. baumannii*. If BlsA interacts with AcoN under blue light avoiding or reducing its ability to bind to target promoter regions, as proposed by the evidence accumulated in this work, then the addition of BlsA to these EMSA assays should reduce the delay observed for the AcoN-DNA probe. DNase protection assays will further characterize the AcoN-DNA binding region. Furthermore, by solving the 3D structures and conducting ultrafast structural dynamic studies of BlsA alone as well as bound to AcoN under blue light, we expect to gain detailed knowledge on structural as well as photochemical aspects of the light signal transduction mechanism.

Finally, we show in this work that quorum network modulators as well as light both regulate the acetoin catabolic cluster. Whether these are independent signals or share totally or partially the signal transduction cascade components is actually under study in our laboratories.

## Data Availability

The datasets generated for this study can be found in GEO, GSE120392.

## Author Contributions

MRT and GLM performed the experiments. PC performed the experiments and collaborated in writing the manuscript. LF-G, LB, and AA performed the experiments and mutant strain. RER and RL-R analyzed the experiments. FF-C and IB analyzed the array studies. BB, RT, ML, and GB developed the RT-PCR experiments. MT designed the experiments. MAM and MT designed the experiments, wrote the manuscript, and provided funding.

## Conflict of Interest Statement

The authors declare that the research was conducted in the absence of any commercial or financial relationships that could be construed as a potential conflict of interest.

## Footnotes

1 http://www.invitrogen.com/content/sfs/manuals/10835031.pdf
