# Supplementary material for: Quorum and Light Signals Modulate Acetoin/Butanediol Catabolism in Acinetobacter spp
Source: Front Microbiol. 2019 Jun 20;10:1376. doi: 10.3389/fmicb.2019.01376 (PMC6595428; doi:10.3389/fmicb.2019.01376)
Supplement: Supplementary file 1 [file Data_Sheet_1.docx]

**SUPPLEMENTARY INFORMATION**

| **STRAIN/PLASMID** | **RELEVANT CHARACTERISTIC** | **SOURCE OR REFERENCE** |
| --- | --- | --- |
| *Acinetobacter sp.strain 5-2Ac02* | Airborne strain | (Barvosa *et al*. 2016) |
| ***A. baumannii*** |  |  |
| ATCC 17978 | Clinical isolate | ATCC |
| ATCC 17978 *ΔabaI* | Generated by mutagenesis using plasmid pMO130-telR | (Castañeda-Tamez *et al.* 2018, Hamad *et al*. 2009) |
| ATCC 17978 *ΔacoN* | Generated by mutagenesis using plasmid pMO130-telR | This study |
| 18 clinical strains | Identified in the "II Spanish Study of *A. baumannii* GEIH-REIPI 2000-2010" | Genbank Umbrella Bioproject PRJNA422585 |
| ATCC 17978 *ΔblsA* | *blsA*::aph derivative of 17978; Km^r^ | (Mussi *et al*., 2010) |
| ATCC 17978 *ΔblsA* pWHBlsA | 17978 *ΔblsA* harboring plasmid pWHBlsA; Km^r^ Amp^r^ | (Mussi *et al*., 2010) |
| ATCC 17978 *ΔblsA* pWH1266 | 17978 *ΔblsA* harboring pWH1266; Km^r^ Tet^r^ Amp^r^ | (Mussi *et al*., 2010) |
| ATCC 17978 *ΔacoN* pWHAcoN | 17978 *ΔacoN* harboring plasmid pWHAcoN; Km^r^ Amp^r^ | This study |
| ATCC 17978 *ΔacoN* pWH1266 | 17979 *ΔacoN* harboring plasmid pWH1266; Km^r^ Tet^r^ Amp^r^ | This study |
| ***E. coli*** |  |  |
| DH5α | Used for DNA recombinant methods | Gibco-BRL |
| ***Saccharomyces cerevisiae*** |  |  |
| Mav 203 strain | MATa, *leu*2-3,112, *trp*1-901, *his*3-D200, *ade*2-101, *gal*4D, *gal*80D, SPAL10::URA3, GAL1::*lac*Z, HIS3UAS GAL1::HIS3, YS2, *can*1R and *cyh*2R | Thermofisher |

| **Plasmids** |  |  |
| --- | --- | --- |
| pMO130-TelR | suicide vector | (Aranda *et al.* 2010, Hamad *et al*. 2009) |
| pBluescript | PCR cloning vector; Ampr | Promega |
| pWH1266 | *E. coli-A. baumannii* shuttle vector; Amp^r^ Tet^r^ | (Hunger *et al.,* 1990) |
| pWHBlsA | pWH1266 harboring wildtype copy of *blsA* from ATCC 17978 expressed under its own promoter; Amp^r^ | (Mussi *et al*., 2010) |
| pWHAcoN | pWH1266 harboring wild-type copy of *acoN* from ATCC 17978 expressed under its own promoter; Amp^r^ | This study |
| pENTR3C | Gateway system entry-vector | Invitrogen- Thermofisher |
| PGAD-T7-GW | Y2H AD-fusion vector, adapted to Gateway System | Clontech, (Cribb and Serra, 2009) |
| PGBK-T7-GW | Y2H DB-fusion vector, adapted to Gateway System | Clontech, (Cribb and Serra, 2009) |

**Table S1.** Bacterial, yeast strains and plasmids used in this study.

| **Gene** | | **SEQUENCE (5'-3')** | **RESTRICTION SITE** | **REFERENCE** |
| --- | --- | --- | --- | --- |
|  |  |  |  |  |
| Up *acoN* | Fow | ATAAGAATGCGGCCGCTAAACTATAAAATCATGTTTCCAACTAGG | Not I | This study |
|  | Rev | CCGGAATTCCGGTACTTGAGTTTCAAAGGAAC | Eco RI |  |
| Down *acoN* | Fow | CCGGAATTCCGGTAGGTATTTAGCTCATCTAAGG | Eco RI | This study |
|  | Rev | CGCGGATCCGCGTAGATGATGTCACTAATTTCAC | Bam HI |  |
| Interno *acoN* | Fow | GCAGCGGTAATATGGTCTG | - | This study |
|  | Rev | AAGAATCACGCCAAAGATGG |  |  |
| Interno *acoN* | Fow | ATTTTATTTGAGCAGCTTAAAGC | - | This study |
|  | Rev | CTTATGCTTTTGTCTGTATCG |  |  |
| P*acoN* | Fow | GGATCCCAAGCATATATGTATAAGTCGAACT | Bam HI | This study |
|  | Rev | GGATCCGTTCCTTTCCAAGCAAATAAGAG | Bam HI |  |
| *blsA*dh | Fow | GGATCCATGAACGTTCGCCTGTGT | - | ([Tuttobene et al., 2018](#_ENREF_35)) |
|  | Rev | CTCGAGTGCTAGAACGGGTTTACTC |  |  |
| *acoN*dh | Fow | GGATCCATGGCAAAGTTAAATTTTGAGTTAG | - | This study |
|  | Rev | CTCGAGACCTAAAGCTTATGCTTTTGTCTGTATC |  |  |

**Table S2.** Primers used in this study.

| ***q*-PCR PRIMERS** | | | | |
| --- | --- | --- | --- | --- |
| **GENE** | | **SEQUENCE (5'-3')** | **PROBE** | **REFERENCE** |
| *abaR* | Fow | AGAGGCGTTACGTTGGACTG | 155/ GAAGGCAA | (Lopez et al., 2017a) |
|  | Rev | CCAAGAATCTGAGCTATTTCTGC |  |  |
| *abaI* | Fow | GGGAACTTCTTTCGGTGGAG | 145/ CAGCGACC | (Lopez et al., 2017a) |
|  | Rev | AACAGCAGCAAGTCGATTATCA |  |  |
| *acoB* | Fow | TGCCAAATAAAAGTTTTCGTAATG | 135/ ATGGCTTC | This study |
|  | Rev | TGCCAAATAAAAGTTTTCGTAATG |  |  |
| *dehydorenase* | Fow | TGGGTGCATCCAATTTCC | 27/CAGGCAGC | This study |
|  | Rev | CCACCGATAACGGTTCAATTA |  |  |
| *rpoB* | Fow | CGTGTATCTGCGCTTGG | 131/ CTGGTGGT | (Fernández-Cuenca et al., 2015) |
|  | Rev | CGTACTTCGAAGCCTGCAC |  |  |
| *rpoB* | Fow | CAGAAGTCACGCGAAGTTGAAGGT |  | (Muller et al., 2017) |
|  | Rev | AACAGCACGCTCAACACGAACT |  |  |
| *recA* | Fow | TACAGAAAGCTGGTGCATGG |  | ([Mussi et al., 2010](#_ENREF_24)) |
|  | Rev | TGCACCATTTGTGCCTGTAG |  |  |
| *acoA* | Fow | AAGATGACGGACTATGCCGTGGAA |  | This study |
|  | Rev | ACGCCGCCAGTCTTTAAGGTTT |  |  |
| *acoB* | Fow | TCAGAAATGCGCCGTGATCCAA |  | This study |
|  | Rev | TTACGCCCAATACACCACCGAAAC |  |  |
| *acoC* | Fow | ACAATGTGCAGCCCAACCACAA |  | This study |
|  | Rev | GGAAATGCGGTGCGTTACGTTT |  |  |

**Table S3**. *q*PCR primers and probes.

| **STRAIN** | **GENOME** | **ACETOIN/BUTANEDIOL CLUSTER (ACC.NUMBER GENBANK/PROTEINS ID)** | | | | | | |
| --- | --- | --- | --- | --- | --- | --- | --- | --- |
|  |  | **AcoA** | **AcoB** | **AcoC** | **AcoD** | **2,3-BDH** | **2,3-BDH** | **AcoN** |
| ***Acinetobacter* sp.** **5-2Ac02** | MKQS00000000 | OFE43540.1 | OFE43541.1 | OFE43542.1 | OFE43543.1 | OFE43544.1 | OFE43545.1 | ND |
| ***A. baumannii* ATCC17978** | CP018664.1 | AUO97_16255 | AUO97_16260 | AUO97_16265 | AUO97_16270 | AUO97_16275 | AUO97_16280 | AUO97_16290 |
| **Ab 155_GEIH-2000^a^** | LJHA00000000 | ODA50985.1 | ODA50984.1 | ODA50983.1 | ODA50982.1 | ODA50981.1 | ODA50980.1 | ODA50978.1 |
| **Ab 158_GEIH-2000 ^a^** | MSMC00000000 | OLV45015.1 | OLV45014.1 | OLV45013.1 | OLV45012.1 | OLV45011.1 | OLV45010.1 | OLV451680.1 |
| **Ab 161_GEIH-2000 ^a^** | MSMB00000000 | OLV49849.1 | OLV49848.1 | OLV49847.1 | OLV49846.1 | OLV49845.1 | OLV49844.1 | OLV49842.1 |
| **Ab 166_GEIH-2000 ^a^** | MSMG00000000 | OLV70189.1 | OLV70190.1 | OLV70191.1 | OLV70192.1 | OLV70193.1 | OLV70194.1 | OLV70196.1 |
| **Ab 169_GEIH-2000 ^a^** | MSMF00000000 | OLV67314.1 | OLV67315.1 | OLV67316.1 | OLV67317.1 | OLV67318.1 | OLV67319.1 | OLV67321.1 |
| **Ab 175_GEIH-2000 ^a^** | MSMI00000000 | OLV81247.1 | OLV81246.1 | OLV81245.1 | OLV81244.1 | OLV81243.1 | OLV81242.1 | OLV81240.1 |
| **Ab 177_GEIH-2000 ^a^** | MSME00000000 | OLV55538.1 | OLV55539.1 | OLV55540.1 | OLV55541.1 | OLV55542.1 | OLV55543.1 | OLV55545.1 |
| **Ab 183_GEIH-2000 ^a^** | MSMJ00000000 | OLV78584.1 | OLV78585.1 | OLV78586.1 | OLV78587.1 | OLV78588.1 | OLV78589.1 | OLV78591.1 |
| **Ab 192_GEIH-2000 ^a^** | MSMH00000000 | OLV63765.1 | OLV63766.1 | OLV63767.1 | OLV63768.1 | OLV63769.1 | OLV63770.1 | OLV63772.1 |
| **Ab 105_GEIH-2010 ^a^** | LJHB00000000 | ODA53436.1 | ODA53435.1 | ODA53434.1 | ODA53433.1 | ODA53432.1 | ODA53431.1 | ODA53429.1 |
| **Ab 33_GEIH-2010 ^a^** | MSMK00000000 | OLV78767.1 | OLV78766.1 | OLV78765.1 | OLV78764.1 | OLV78763.1 | OLV78762.1 | OLV78760.1 |
| **Ab 49_GEIH-2010 ^a^** | MSMM00000000 | OLV86121.1 | OLV86122.1 | OLV86123.1 | OLV86124.1 | OLV86125.1 | OLV86126.1 | OLV86128.1 |
| **Ab 54_GEIH-2010 ^a^** | MSML00000000 | OLV85204.1 | OLV85205.1 | OLV85206.1 | OLV85207.1 | OLV85208.1 | OLV85209.1 | OLV85211.1 |
| **Ab 76_GEIH-2010 ^a^** | MSLY00000000 | OLV41736.1 | OLV41735.1 | OLV41734.1 | OLV41733.1 | OLV41732.1 | OLV41731.1 | OLV41737.1 |
| **Ab 103_GEIH-2010 ^a^** | MSLX00000000 | OLV35374.1 | OLV35375.1 | OLV35376.1 | OLV35377.1 | OLV35378.1 | OLV35379.1 | OLV39499.1 |
| **Ab 104_GEIH-2010 ^a^** | MSMA00000000 | OLV45315.1 | OLV45314.1 | OLV45313.1 | OLV45312.1 | OLV45311.1 | OLV45310.1 | OLV45316.1 |
| **Ab 121_GEIH-2010 ^a^** | MSLZ00000000 | OLV34644.1 | OLV34645.1 | OLV34646.1 | OLV34647.1 | OLV34648.1 | OLV34649.1 | OLV34651.1 |
| **Ab 122_GEIH-2010 ^a^** | MSMD00000000 | OLV53193.1 | OLV53194.1 | OLV53195.1 | OLV53196.1 | OLV53197.1 | OLV53198.1 | OLV53200.1 |

**Table S4.** Genomes and Proteins in the acetoin/butanediol cluster. Genbank database of *A. baumannii* strains isolated in the "II Spanish Study of *A. baumannii* GEIH-REIPI 2000-2010 " (Genbank Umbrella Bioproject PRJNA422585). ND: Not detected in the genome.


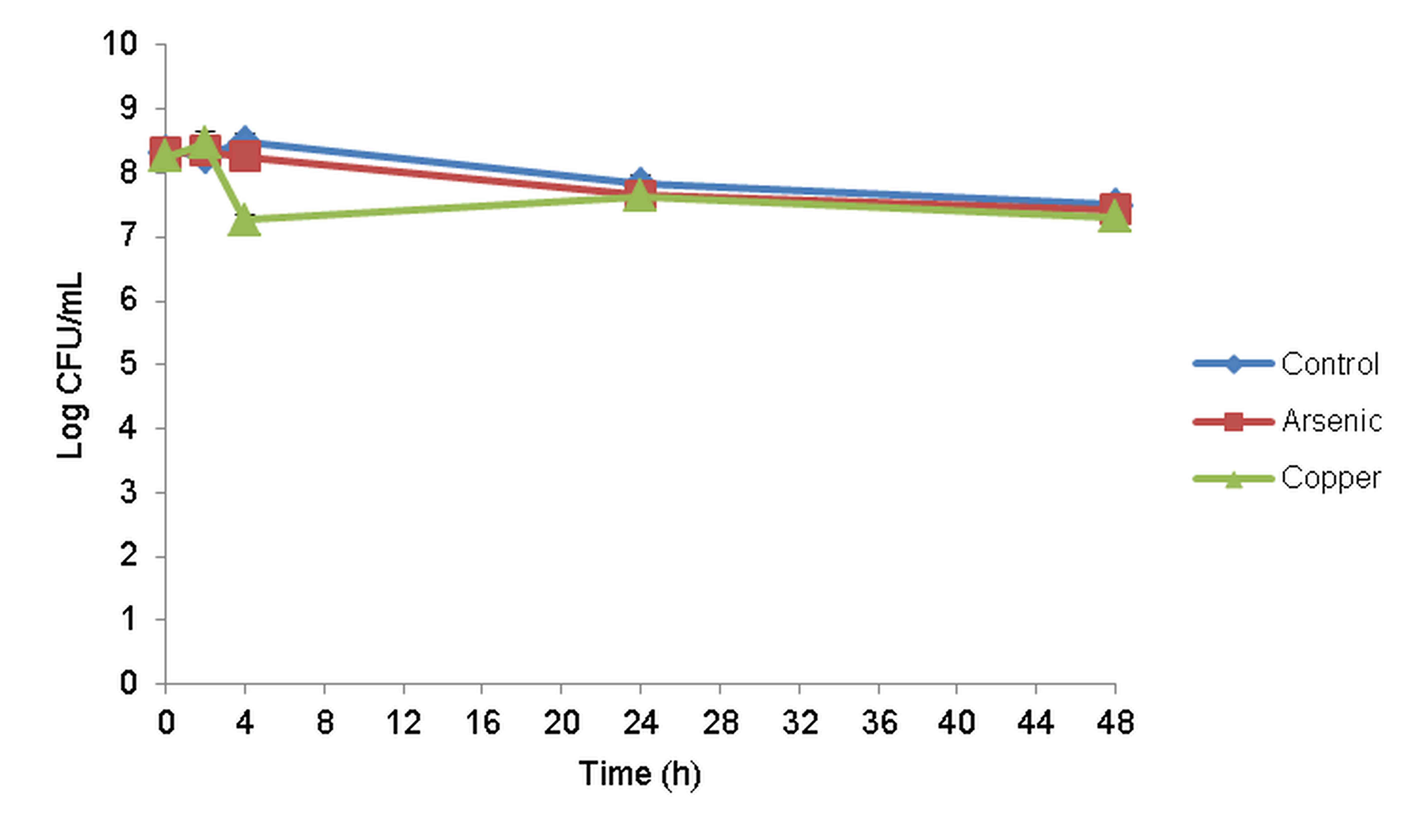


**Figure S1.** *Acinetobacter* sp. strain 5-2Ac02 is resistant to arsenic and copper. Growth curves of *Acinetobacter* sp. strain 5-2Ac02 in low LB browth in the absence (control) or in the presence of arsenic 1024 µg/ml and copper 133 µg/ml at 37ºC.

**
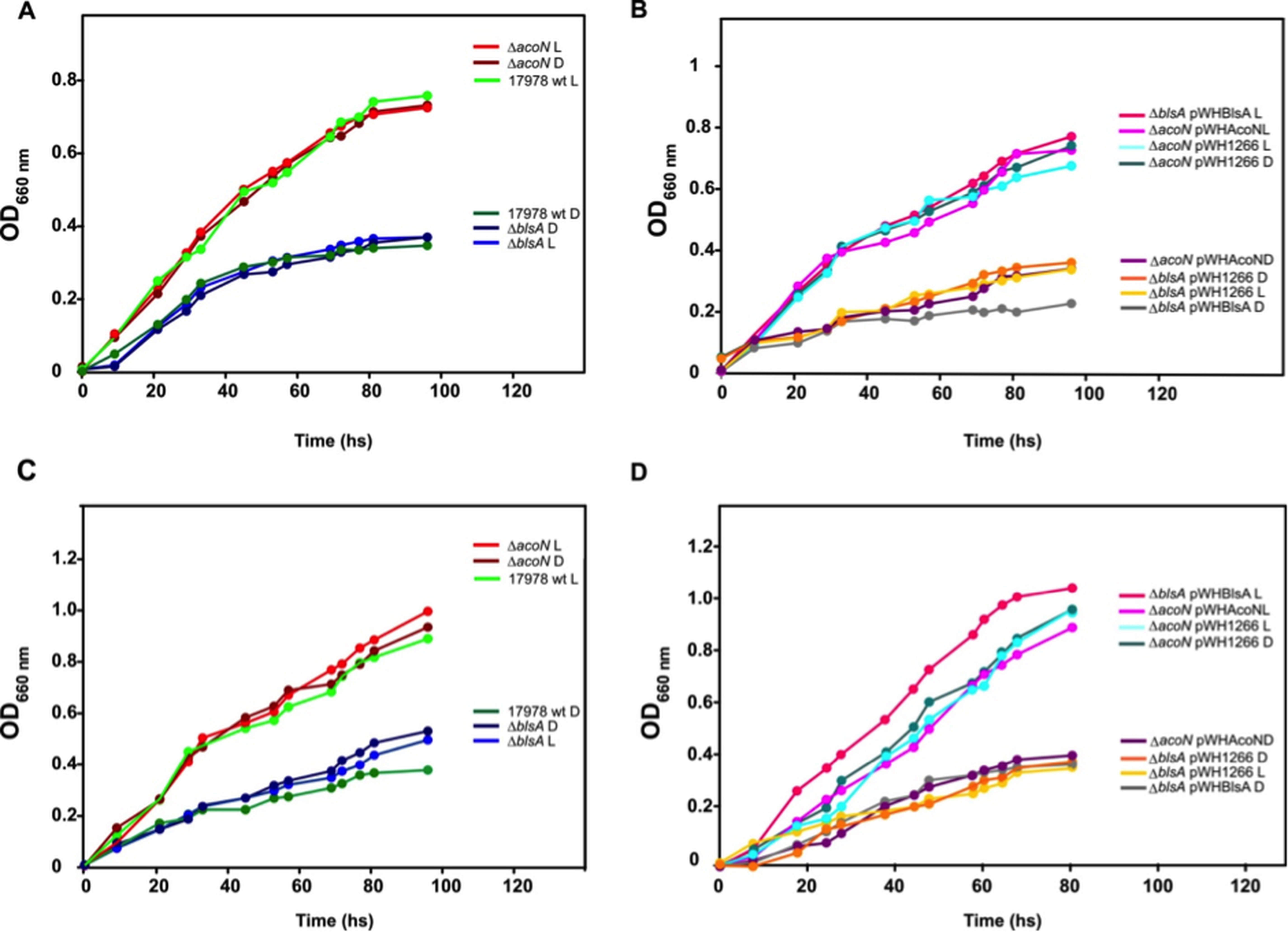
**

**Figure S2.** Light modulates acetoin catabolism at moderate temperatures in *A. baumannii* ATCC 17978. **A and B.** Growth curves in M9 minimal medium supplemented with acetoin 10 mM as sole carbon source of *A. baumannii* ATCC 17978 wild-type and derivative strains, incubated stagnantly at 23°C under blue light (L) or in the dark (D). **C and D.** Growth curves in M9 minimal medium supplemented with acetoin 15 mM as sole carbon source of *A. baumannii* ATCC 17978 wild-type and derivative strains, incubated stagnantly at 23°C under blue light (L) or in the dark (D).
